# microRNA targeting of the P2X7 purinoceptor opposes a contralateral epileptogenic focus in the hippocampus

**DOI:** 10.1038/srep17486

**Published:** 2015-12-03

**Authors:** Eva M. Jimenez-Mateos, Marina Arribas-Blazquez, Amaya Sanz-Rodriguez, Caoimhin Concannon, Luis A. Olivos-Ore, Cristina R. Reschke, Claire M. Mooney, Catherine Mooney, Eleonora Lugara, James Morgan, Elena Langa, Alba Jimenez-Pacheco, Luiz Fernando Almeida Silva, Guillaume Mesuret, Detlev Boison, M. Teresa Miras-Portugal, Michael Letavic, Antonio R. Artalejo, Anindya Bhattacharya, Miguel Diaz-Hernandez, David C. Henshall, Tobias Engel

**Affiliations:** 1 Department of Physiology & Medical Physics, Royal College of Surgeons in Ireland, Dublin, Ireland; 2 Department of Toxicology and Pharmacology, Faculty of Veterinary Medicine, Universidad Complutense de Madrid, Madrid, Spain; 3 Robert S. Dow Neurobiology Laboratories, Legacy Research Institute, Portland, OR, USA; 4 Department of Biochemistry, Faculty of Veterinary Medicine, Universidad Complutense de Madrid, Madrid, Spain; 5 Janssen Research & Development, LLC, Neuroscience, 3210 Merryfield Row, San Diego, CA 92121, San Diego, USA

## Abstract

The ATP-gated ionotropic P2X7 receptor (P2X7R) modulates glial activation, cytokine production and neurotransmitter release following brain injury. Levels of the P2X7R are increased in experimental and human epilepsy but the mechanisms controlling P2X7R expression remain poorly understood. Here we investigated P2X7R responses after focal-onset status epilepticus in mice, comparing changes in the damaged, ipsilateral hippocampus to the spared, contralateral hippocampus. P2X7R-gated inward currents were suppressed in the contralateral hippocampus and *P2rx7* mRNA was selectively uploaded into the RNA-induced silencing complex (RISC), suggesting microRNA targeting. Analysis of RISC-loaded microRNAs using a high-throughput platform, as well as functional assays, suggested the P2X7R is a target of microRNA-22. Inhibition of microRNA-22 increased P2X7R expression and cytokine levels in the contralateral hippocampus after status epilepticus and resulted in more frequent spontaneous seizures in mice. The major pro-inflammatory and hyperexcitability effects of microRNA-22 silencing were prevented in *P2rx7*^*−/−*^ mice or by treatment with a specific P2X7R antagonist. Finally, *in vivo* injection of microRNA-22 mimics transiently suppressed spontaneous seizures in mice. The present study supports a role for post-transcriptional regulation of the P2X7R and suggests therapeutic targeting of microRNA-22 may prevent inflammation and development of a secondary epileptogenic focus in the brain.

Focal epilepsies such as temporal lobe epilepsy (TLE) are the most common and intractable seizure disorders in adults^1^. The cell and molecular mechanisms underlying TLE remain incompletely understood. The hippocampus from TLE patients and animal models of epilepsy often displays gliosis and markers of inflammation. This may contribute to the pathogenesis and maintenance of the epileptic state by altering neuronal and network functions thereby changing the balance between excitation and inhibition in the brain^2^,^3^. Intriguingly, experimental and human studies also show neuroinflammation extends to contralateral brain regions^4^,^5^ which may additionally influence epileptogenesis^6^. Improved understanding of the molecular mechanisms controlling neuroinflammation within the ipsilateral seizure focus and beyond may yield novel targets for the treatment or prevention of epilepsy.

Adenosine triphosphate (ATP) functions as a glio- and neuro-transmitter to modulate brain excitability and neuroinflammation^7^. The fast effects of extracellular ATP are mediated via the P2X class of ionotropic receptor which gates depolarizing sodium and calcium entry into cells^8^. Among the seven members of the P2X family, there has been most focus on the P2X7 receptor (P2X7R) in neurological diseases^8^. The P2X7R is activated only under conditions of pathologically high extracellular ATP such as during seizures and brain injury, displays non-desensitizing currents and has possible direct cell-killing effects^8^,^9^,^10^. The downstream consequences of P2X7R signaling include microglia activation and stimulating the release of the pro-convulsive inflammatory cytokine interleukin 1β (IL-1β)^11^,^12^,^13^. There is also evidence that the P2X7R is expressed by neurons and modulates neurotransmitter release^14^,^15^. P2X7R levels are increased in experimental models of epilepsy and in resected brain tissue from pharmacoresistant TLE patients^11^,^16^,^17^. Recent work showed that pharmacologic blockade or genetic ablation of the P2X7R reduces seizure severity during prolonged seizures (status epilepticus) in rodents^17^,^18^,^19^,^20^. The P2X7R therefore represents an attractive target for the treatment of seizures or attendant neuroinflammation^10^.

The molecular mechanisms controlling P2X7R expression in the brain are largely unknown. Recent work in non-CNS cells showed that P2X7R expression is regulated by microRNAs (miRNA)^21^,^22^; small ~19–24 nt non-coding RNAs that function post-transcriptionally to regulate gene expression in cells^23^. The most common effect of miRNAs is to reduce protein levels of their targets^24^. To function, miRNAs are uploaded to the RNA induced silencing complex (RISC) where Argonaute 2 (Ago2) facilitates base-pairing to target mRNAs, resulting in translational repression or degradation of the mRNA^25^. Both experimental and human TLE are associated with altered expression of miRNA within the seizure focus^26^ and recent studies showed that manipulation of miRNAs controlling neuronal microstructure had potent effects on convulsive thresholds^27^,^28^. It is likely, however, that miRNAs control other substrates of epileptogenesis such as ion channels and neuroinflammation^2^,^29^,^30^. In the present study, we used a focal-onset model of status epilepticus in mice^31^,^32^ to explore molecular mechanisms controlling P2X7R expression. We identify a miRNA targeting the P2X7R in the contralateral hippocampus that functions to suppress neuroinflammatory signaling and epilepsy.

## Materials and Methods

### Status epilepticus in mice

All animal experiments were performed in accordance with the European Communities Council Directive (86/609/EEC) and were reviewed and approved by the Research Ethics Committee of the Royal College of Surgeons in Ireland, under license from the Department of Health, Dublin, Ireland. Adult male C57Bl/6 mice (20–25 g) were obtained from Harlan (Oxon, Bicester, U.K.). P2X7R reporter mice [Tg(*P2rx7*-EGFP)FY174Gsat/Mmcd, stock 011959-UCD] were from U.S. National Institutes of Health Mutant Mouse Regional Resource Centers and granted by Dr. M. Nedergaard (University of Rochester, Rochester, NY, USA). P2X7R-deficient mice, in which the carboxyl-terminal coding region of the *P2rx7* gene is disrupted by a targeting vector containing a neomycin resistance gene driven by the mouse phosphoglycerate kinase promoter, were obtained from the Jackson Laboratory (B6.129P2-P2rx7^tm1Gab^/J, stock 005576; Jackson Laboratory, Bar Harbor, ME, USA).

Status epilepticus was induced as previously described^27^,^31^. Under isoflurane anesthesia, mice were equipped with skull-mounted recording screws and injection cannula (Bilaney Consultants, Sevenoaks, Kent, UK). Status epilepticus was then triggered in freely-moving awake mice by intra-amygdala microinjection of kainic acid (0.3 μg) (Sigma-Aldrich, Ireland). Control animals received intra-amygdala vehicle (phosphate-buffered saline (PBS)). After 40 min, all mice received lorazepam (6 mg/kg, i.p.) to curtail seizures and reduce morbidity and mortality. For combined intrahippocampal and cortical recordings, mice were implanted with a bipolar electrode (Bilaney Consultants Ltd), into the dorsal CA3 subfield of the hippocampus (Coordinates from bregma; AP = −2.25 mm, L = −0.9 mm, V = −1.94 mm)^33^.

Mice were euthanized at various time points after status epilepticus or drug administration. Time points were chosen to capture early post-status epilepticus changes (8, 12 and 24 h), the time of spontaneous seizure onset (72 h), and during the chronic phase of epilepsy (14 days) in the model^32^. Animals were given a pentobarbital overdose and perfused with ice-cold saline to remove intravascular blood components. Brains for molecular and biochemical work were microdissected over dry ice. For histology, mice were perfusion-fixed with paraformaldehyde (4%) or brains fresh-frozen in 2-methylbutane (at −30 °C).

### *In vivo* drug treatments

Coordinates for i.c.v. injections were bregma: AP = −0.3 mm, L = −1.0 mm, V = −2.0 mm^33^. Antagomirs were from Exiqon (locked nucleic acid (LNA)- and 3′-cholesterol modified oligonucleotides). The miR-22 antagomir sequence was CTTCAACTGGCAGCT and scrambled was ACGTCTATACGCCCA. Mice received 2 μL infusion of 0.5 nmol antagomir/scrambled in artificial cerebrospinal fluid (aCSF) (Harvard Apparatus UK, Kent, UK). To overexpress miR-22 we used chemically-modified double-stranded RNAs (mirVana™ mimics; Life technologies). Nanoparticles of the mimics or non-targeting controls (range 0.3 pmol–1 nmol) were generated using Invivofectamine®2.0 (Life technologies) following manufacturer instructions. Two microliters of the complex was injected i.c.v. into mice. For systemic injections during long-term studies, JNJ-47965567 or minocycline (both 30 mg/kg) or vehicle (DMSO or PBS, respectively) were injected intraperitoneal twice daily (with first injection 1 h after intra-amygdala kainic acid injection).

### EEG analysis

EEG was analyzed as before^18^,^27^ using TWin® software with additional frequency/amplitude analysis of EEG by LabChart Pro v7 software (ADInstruments Ltd, Oxford, UK). Epilepsy monitoring was performed via implanted EEG telemetry units, as previously described^27^. EEG data were acquired with EEG transmitters (Model:F20-EET, Data Systems International, St. Paul, MN, USA) configured to record 2-channel EEG that were skull-affixed over dorsal hippocampi/temporal cortex. Continuous (24 h/day) EEG data were collected for up to 14 days. EEG data were reviewed and manually scored by an observer unaware of experimental treatment with epileptic seizures defined as high frequency (>5 Hz) high amplitude (>2 × baseline) polyspike discharges of >5 s duration.

### Behavior analysis

Object location experiments were performed to test hippocampal function, as described previously^34^. On the training day, mice were placed in an arena for four 10-min sessions with an inter-session interval of 3 min, during which mice were returned to their home cages. The first session consisted of a context habituation period. During the next three sessions, mice were placed in the training arena with two distinct objects. Twenty-four hours after training, mice were placed in the original training arena for 10 min with one object moved to a new location. Exploration of objects was measured manually by stopwatch and was defined as the amount of time mice were oriented towards an object with its nose within 1 cm of it, scored by an experimenter blinded to treatment.

### Electrophysiological recordings in brain slices and N2a cells

Whole-brains were sagitally sectioned to obtain 300 μm-thick slices, which were kept in saline solution continuously bubbled with carbogen (95% O_2_/5% CO_2_) at room temperature. Brain slices fixed with a nylon grid or N2a cells plated to glass coverslips (10^4^ cells/mL; 24–48 h after transfection) were transferred to a submersion chamber attached to the stage of an upright microscope (Olympus BX51W1; UK) and superfused with a saline solution. Cells were viewed under a 63 × water immersion objective, and fluorescence illumination and a DL-604 OEM camera (Andor Technology, EU) were used to visualize EGFP-cells in the dentate gyrus region of the hippocampus and transfected N2a cells. Electrophysiological recordings were performed with an EPC10/2 patch-clamp amplifier using PatchMaster software (HEKA Electronic, Lambrecht, Germany). Patch pipettes had a final resistance of 5–6 MΩ when filled with a solution containing (in mM) 140 N-Methyl-D-glucamine (NMDG+), 5 EGTA, 3 MgCl2, and 10 HEPES (pH 7.2, adjusted with HCl; ≈290 mOsm). Membrane currents, measured in whole-cell configuration were filtered at 3 kHz and sampled at 10 kHz. Once electrical access to the cytoplasm was established, cells were held at a voltage (Vh) of −70 mV. Series resistance (5–10 MΩ) was compensated by 80% and monitored throughout the experiment together with the cell membrane capacitance. Drug application was started at least 10 min after obtaining the whole-cell configuration, and experiments in which series resistance changed by more than 20% or holding current exceeded 20 pA were not analyzed. All recordings were obtained at room temperature. Currents were activated by BzATP (100 μM; Sigma-Aldrich) applied for 3 s at 2 min intervals onto the cell under investigation by means of a pneumatic drug ejection system (PDES-02DX, NPI Electronic GmbH, Germany). A-438079 (A43; 10 μM; Sigma-Aldrich) was applied from a separate glass pipette 2 min before and during agonist administration.

### Histopathology

Seizure-induced neuronal damage was analyzed on 12 μm coronal sections at the level of medial hippocampus (AP = −1.70 mm)^33^ using Fluoro-Jade B (FJB) (Millipore Ireland B.V.) as described^18^,^27^. To mark potentially reversible injury we performed silver staining. Briefly, sections were processed with FD Neuro Silver kit II (FD Neurotechnologies Inc., Baltimore, MD), following the manufacturers’ protocol. For immunohistochemistry, sections were incubated overnight with antibodies against NeuN (1:400, Cat n: MAB377. Millipore), GFAP (1:400, Cat n: G4546. Sigma-Aldrich), Neuropeptide Y (NPY, 1:1000, Cat n: HPA044572. Sigma-Aldrich), GFP (1:500, Cat n: AKR-020. Cell biolabs), P2X7R (1:200, Cat n: APR-004. Alomone labs), c-Fos (1:50, cat n: sc7202. Santa Cruz Biotechnology), ADK (1:3000. Custom made) and Iba1 (1:400, Cat n: 019-19741. Wako). After washing, sections were incubated with fluorescent secondary antibodies (BioSciences Ltd.) or DAB stained (VectorLabs). Synaptic reorganization was assessed in NPY-stained sections using a scale adapted from methods for mossy fiber sprouting assessment^27^. Staining was examined using a Leica DFC310FV camera attached to a Leika 4000B microscope under fluorescence or white light. For density analysis of microglia two photomicrographs (40 × lens) from the DG/hilus were analyzed using Image J software and signal was the mean of both areas. Counts were the average of two adjacent sections scored by an observer blinded to experimental treatment.

### RNA extraction and real time PCR

Total RNA was extracted using the Trizol method^27^,^35^. The quality and quantity of RNA were measured using a Nanodrop Spectrophotometer (Thermo Scientific) and samples with an absorbance ratio at 260/280 between 1.8–2.2 were considered acceptable. RNA degradation was not assessed. RNA dilutions were made up in nuclease-free water. For analysis of mRNA expression, 1 μg of total RNA was used to generate cDNA by reverse transcription using Superscript II Reverse Transcriptase enzyme (Invitrogen). Quantitative real-time PCR was performed using a LightCycler 1.5 (Roche Diagnostics) in combination with QuantiTech SYBR Green PCR kit (Qiagen Ltd) as per manufacturer’s protocol and 1.25 μM of primer pair used. Data were represented as 2^−ΔΔCT^ and normalized to expression of β-actin. Primers used: *Adenosine kinase* (Forward (F): gaggcttgtcagagacagt, Reverse(R): cttcacacaagggcgagatg); *Arc* (F: agcagcagacctgacatcct, R: gtgatgccctttccagacat); *c-fos* (F: ggaattaacctggtgctgga, R: cattcagaccacctcgacaa); *GFP* (F: acgtaaacggccacaacttc, R: aagtcgtgctgcttcatgtg); *Iba1* (F: tggaggggatcaacaagcaa, R: accccaagtttctccagcat); *NEFH* (F: agtggttccgagtgaggttg, R: ctgctgaatagcgtcctggt); *Il1*β (F: tgaagttgacggaccccaaa, R: agcttctccacagccacaat); *P2rx7* (F:actggcaggtgtgttccata, R: ttggcaagatgtttctcgtg); *SP1* (F: tcataccaggtgcaaaccaa, R: aggtgatgttcccattcagg); *Gfap* (F: agaaaaccgcatcaccattc, R: tcacatcaccacgtccttgt); *Tnf*α (F: ctcttcaagggacaaggctg, R: cggactccgcaaagtctaag) and β*-actin* (F: gggtgtgatggtgggaatgg, R: ggttggccttagggttcagg).

For miRNA analysis, reverse transcription for individual qPCR was carried out using 500 ng of total RNA and the High-Capacity Reverse Transcription Kit (Applied Biosystems). RT specific primers for miR-22, miR-19a, miR-92, miR-150 and miR-181 (Applied Biosystems) were used for all miRNA reverse transcription. Individual qPCRs were carried out on the QuantStudio 12k Flex System (Applied Biosystems) using miR-22, miR-19a, miR-150 and miR-181 specific Taqman microRNA assays (Applied Biosystems). RNU6B was used for normalization of miRNA expression due to its size and abundance in the tested material, manufacturer recommendations, and previous use of this small RNA for normalizing miRNA expression in brain tissue studies^27^,^36^. A relative fold change in expression of the target gene transcript was determined using the comparative cycle threshold method (2^−ΔΔCT^).

### Western blotting

Synaptoneurosomes were prepared from mouse hippocampus as described^17^ and western blot analysis was performed as previously^27^. Hippocampal proteins were separated by SDS-PAGE and immunoblotted using the following primary antibodies: P2X7R (1:200, Cat n: APR-004, Alomone labs), IL-1β (1:2000, Cat n: ab9772. Abcam), synaptophysin (1:1000, Cat n: S5768. Sigma-Aldrich), α-tubulin (1:2000, Cat n: T6199. Sigma-Aldrich) and β-actin (1:2000, Cat n: A5441. Sigma-Aldrich). Membranes were then incubated with horseradish peroxidase-conjugated secondary antibodies (Jackson ImmunoResearch, Plymouth, PA, USA) and bands visualized using Supersignal West Pico Chemiluminescence Substrate (Pierce, Rockford, IL, USA). Images were captured using a Fuji-Film LAS-300 (Fuji, Sheffield, UK), and densitometry was performed using AlphaEaseFC4.0 gel-scanning integrated optical density software (Alpha Innotech, San Leandaro, CA, USA).

### *In situ* hybridization

Techniques were as previously described^27^,^34^. Using RNAse free solutions, slides were treated with 0.25% acetic anhydride/0.1 M triethanolamine, followed by 5μg/ml proteinase K. Next, slides were rinsed in hybridization buffer. The probes to detect miR-22 or the antagomir were 2′-O,4′-C methylene bicyclonucleoside monomer-containing oligonucleotides (LNA-modified. Exiqon). Sequence for the probes: anti-miR-22: CTTCAACTGGCAGCTT/3Dig_N and anti-antagomir; 5DigN/AGCTGCCAGTTGAAG/3Dig_N. A scrambled probe was also included to assess non-specific binding (Exiqon). Probes were incubated 1:200 in hybridization buffer overnight at 60 °C. Sections were washed and then incubated with anti-DIG-PA antibody (1:1000, Roche). The following day sections were washed again and then reacted with colour substrate solution (CSS: Nitroblue tetrazolium/BCIP stock solution (Roche). Slides were then rinsed, mounted with medium and coverslipped.

### Analysis of RISC by immunoprecipitation of Ago2

CA3-DG subfields from individual mice were obtained by microdissection under a microscope and pools of 4–5 were homogenized in an immunoprecipitation buffer and centrifuged. Five micrograms of Ago2 antibody (Cell Signaling Technology, Danvers, MA, USA), was added to 400 μg of the supernatant, vortex-mixed and incubated overnight at 4 °C. Then, 20 μl of 50% Protein-A/G-agarose bead solution (Santa Cruz Biotechnology, Heidelberg, Germany) was added, mixed and incubated. The beads were centrifuged and the pellet washed followed by Trizol extraction^27^.

### miRNA profiling by OpenArray

100 ng of RNA purified from the Ago2 immunoprecipitation was processed by reverse transcriptase and pre-amplification steps following the manufacturer’s protocol (Applied Biosystems). The pre-amplification reaction was mixed with TaqMan OpenArray Real-Time PCR Master mix (1:1). The mix was loaded onto the OpenArray rodent panel (750 mouse/rat miRNAs) and ran using a QuantStudio 12 K Flex PCR (Life Technologies). The number of miRNA identified in each sample showed good consistency: 338, 352, 370 and 337 in control contralateral, control ipsilateral, kainic acid contralateral and kainic acid ipsilateral, respectively. Any microRNA that was not expressed (i.e. Ct value < 35) in at least one sample was removed, leaving 409 of the original 750 miRNA. The mean Ct (and standard deviation) of the Ct values was: 24.74 (3.83), 24.28 (3.81), 23.34 (3.94) and 24.38 (3.96) for control contralateral, control ipsilateral, kainic acid contralateral and kainic acid ipsilateral respectively.

There are no universally invariant miRNA or any other small RNA molecule that has been found to date suitable for normalization^37^. Therefore, the data was normalised using a global normalisation method where each Ct value was normalised to the geometric mean of all Ct values in a sample, as implemented in the R Bioconductor package “HTqPCR”^38^. The heat map was generated using heatmap.2 in the R statistical analysis programme. The OpenArray sequences and miRNA names were mapped to the mature human sequences from miRBase^39^ version 21.

### Cell culture

N2a cells were cultured in supplemented DMEM medium (Invitrogen) and seeded at 25000 cell/cm^2^. Primary cultures of hippocampal neurons were prepared from E18 embryonic mice as described^27^. Neurons were plated onto poly-L-lysine and laminin bed and maintained in Neurobasal medium supplemented with B-27 and N2 (Gibco), at 37 °C in a humidified atmosphere with 5% (v/v) CO_2_ for 7–10 days. Primary astrocyte and microglial cell cultures were isolated from hippocampi (astrocyte) or hippocampi/cortex (microglia) of P0 neonatal mice pups. After microdissection, hippocampi were triturated and passed through a 40 μm nylon cell strainer (BD Falcon) and cells were cultured for a minimum of 14 days with DMEM-F12 for astrocytes or supplemented DMEM-F12 (10 ng/ml GM-CSF and 20 ng/ml CSF (R&D systems)) for microglia. Cell culture purity was determined by measurement of appropriate marker genes.

### Bioinformatics

Predicted targets of mmu-miR-22 were identified using miRanda and microRNA.org and those with good mirSVR scores were selected, which generated 2902 targets. The mouse proteome (Uniprot; 43,537 proteins) was interrogated for proteins with the term “ion channel” and this identified 468. We then searched the 2902 predicted targets of mmu-miR-22 against the 468 mouse proteins with the term “ion channel” and found 15 proteins, which were *Ank3*, *Arrb1*, *Clcc1*, *Cav3*, *Cnga2*, *Itpr1*, *Mylk*, *P2rx7*, *Ryr3*, *Tmc3*, *Trpc5*, *Trpm5*, *Trpm6*, *Trpm7* and *Unc80*.

### Data analysis

All data are presented as mean ± standard error of the mean. Two group comparisons were made using unpaired Student’s t-test, while multi-group comparisons were made using one and two-way analysis of variance (ANOVA) followed by appropriate post hoc testing (StatView). Significance was accepted at *P* < 0.05.

## Results

### P2X7R function in the hippocampus after status epilepticus

**Figure 1:**
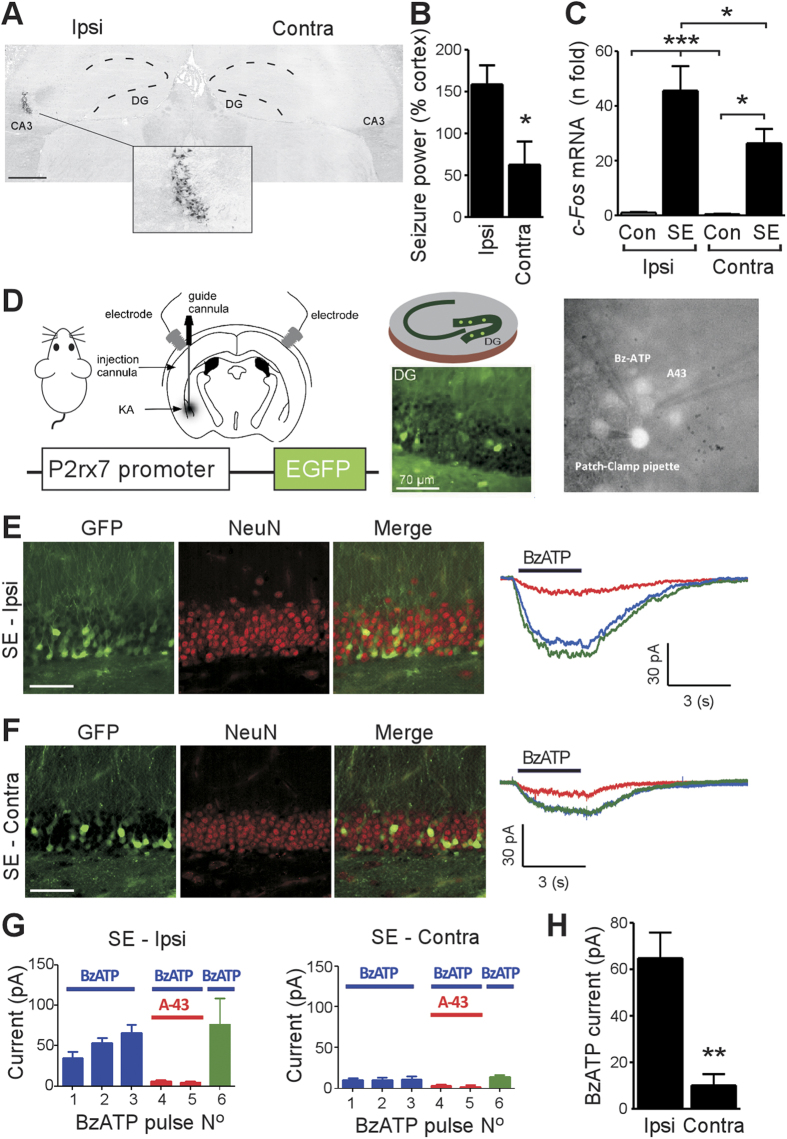
Reduced P2X7R responses in the contralateral hippocampus after focal-onset status epilepticus. (**A**) Representative Fluoro-Jade B staining of the hippocampus 72 h after status epilepticus (SE) (from *n* = 5 mice). Note, ipsilateral (Ipsi) CA3 lesion (see inset box) while sparing the dentate gyrus (DG, outlined) and contralateral (Contra) side. Image was created by stitching photomicrographs from the same brain section. Bar, 500 μm. (**B**) Intrahippocampal EEG recordings show seizure power is reduced on the contralateral side (*n* = 5/group). (**C**) Levels of the activity-regulated gene *c-Fos* in the contralateral hippocampus 4 h after SE compared to the ipsilateral hippocampus (*n* = 4–5/group). (**D**) Schematic showing reporter mouse and method for triggering SE by intra-amygdala kainic acid (original drawing by author DC Henshall). Cartoon (right) shows *ex vivo* slice preparation and view of recording field within the DG. (**E,F**) Representative images of EGFP-positive cells within the Ipsi and Contra DG layer (neurons; red) 12 h after SE. EGFP expression is under the control of the *P2rx7* promoter. Scale bar, 60 μm. Representative traces (right) show patch-clamp recordings from EGFP-positive cells from the DG in response to the P2X7R ligand BzATP (100 μM) (blue line) after SE. Inward currents in EGFP-positive cells were reversibly blocked by co-administration of the P2X7R antagonist A-438079 (10 μM) (red line). Currents could be re-elicited by BzATP after antagonist washout (green line). (**G**) Quantification of patch clamp recordings from EGFP-positive cells from the Ipsi and Contra DG. BzATP (100 μM) was repeatedly applied for 3 s intervals. Inward currents in EGFP-positive cells were reversibly blocked by A-438079 (10 μM) (red). Green bar shows recovery of responses to BzATP after antagonist washout (*n* = 11 cells from 6 different mice). Note, similar numbers of EGFP-positive cells between each side but only minimal BzATP currents are evoked on the Contra side (*n* = 11 cells from 6 different mice). (**H**) Quantification of BzATP evoked currents from EGFP-positive cells in the contralateral compared to the Ipsi hippocampus. Data represent averaged values of three consecutive responses to BzATP. **p < 0.01; compared to Ipsi.

A schematic of the experimental design is provided in Supplementary Fig. S1. Status epilepticus was triggered in mice by intra-amygdala microinjection of kainic acid. In this model, prolonged seizures lead to neuronal death and gliosis that is confined to the ipsilateral hippocampus while the contralateral hippocampus is spared damage^31^,^40^. Confirming this unilateral feature, analysis of tissue sections stained for Fluoro-Jade B revealed irreversible neuronal death in the ipsilateral but not contralateral hippocampus after status epilepticus (Fig. 1A). Intra-hippocampal EEG recordings determined that the contralateral hippocampus was recruited during status epilepticus but seizure severity was lower compared to the ipsilateral side (Fig. 1B). Up-regulation of the neuronal activity marker gene *c-Fos* was also lower in the contralateral compared to the ipsilateral hippocampus (Fig. 1C). Thus, the contralateral hippocampus experiences seizures in this model but does not manifest permanent tissue damage.

To study P2X7R function in this model, we performed patch-clamp recordings from *ex vivo* brain slices. Since the P2X7R is not ubiquitously expressed, we identified positive cells using a transgenic mouse in which enhanced green fluorescent protein (EGFP) is under the control of the *P2rx7* promoter^18^ (Fig. 1D). Thus, transcription of the *P2rx7* transcript is accompanied by the appearance of EGFP in the cell. Twelve hours after status epilepticus, multiple EGFP-positive cells with the morphological appearance of neurons were present in the ipsilateral dentate granule cell layer in reporter mice (Fig. 1E). P2X7R channel properties are distinct from other P2X receptors and feature non-desensitizing inward currents that increase with repeated agonist stimulation^9^. Consistent with functional P2X7Rs in the ipsilateral hippocampus, repeated application of the P2X7R agonist BzATP induced increasing depolarizing inward currents during patch-clamp recordings from EGFP-positive cells (Fig. 1E,G). Co-application of the selective P2X7R antagonist A-438079 reversibly abolished BzATP-induced inward currents (Fig. 1E,G).

In the contralateral hippocampus we observed similar numbers of EGFP-positive cells in the granule cell layer 12 h after status epilepticus (Fig. 1F). However, patch-clamp recordings from EGFP-positive cells in the contralateral granule cell layer detected only weak currents in response to BzATP applications (Fig. 1F,G). Quantitative comparison of BzATP-evoked currents in EGFP-positive cells confirmed that responses in contralateral slices were ~80% lower than the ipsilateral side (Fig. 1H).

### MicroRNA-22 targets the P2X7R in the contralateral hippocampus

**Figure 2:**
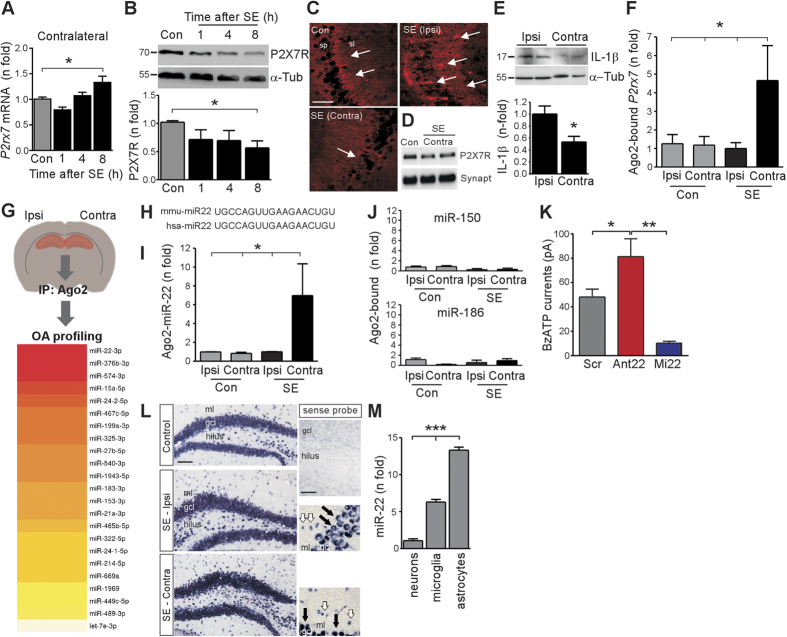
miR-22 is uploaded to the RISC in the contralateral hippocampus. (**A**) *P2rx7* mRN**A** levels in the Contra hippocampus after SE (*n* = 5/group). (**B**) Immunoblot (*n* = 1/lane) and graph showing reduced P2X7R protein in the Contra hippocampus (*n* = 4/group). α-Tubulin (α-Tub) is included as a guide to loading. (**C**) P2X7R immunoreactivity in the CA3 subfield. Note the reduced staining in the Contra hippocampus 8 h after SE (arrows). Sp, stratum pyramidale; sl, stratum lucidum. Scale bar, 50 μm. (**D**) Immunoblot (*n* = 3/lane) showing P2X7R protein at 8 h within synaptodendritic fraction. Synaptophysin (Synapto) is used as loading control. (**E**) Immunoblot and graph showing mature IL-1β in the hippocampus after SE (8 h; *n* = 4/group). (**F**) qPCR analysis of Ago2-eluted *P2rx7* mRNA, 8 h after SE (*n* = 5/group). (**G**) RISC profiling (original drawing by author DC Henshall). Heat map lists miRNAs in order of abundance in the Contra hippocampus. (**H**) Mouse (mmu) and human (hsa) miR-22-3p sequence. (**I**) qPCR analysis of miR-22 from Ago2 pull-downs 8 h after SE (*n* = 6; each sample was a pool of 2 hippocampi). (**J**) miR-150 and miR-186 levels in Ago2 pull-downs 8 h after SE (*n* = 5/group). (**K**) Patch clamp recordings of P2X7R agonist responses in N2a cells transfected with scramble (Scr), antagomir-22 (Ant22) and miR-22 mimic (Mi22) (*n* = 4–5/group). (**L**) *In situ* hybridization showing miR-22 in control brain and 8 h after SE. Black arrows, granule neurons; miR-22 was also present in glia-like cells (white arrows). Panels on right shows a staining control and higher-power images of miR-22 staining after SE. Scale bar, 100 μm. gcl, granule cell layer; ml, molecular layer. (**M**) miR-22 expression in cultures of neurons, microglia and astrocytes (from *n* = 3 experiments). *p < 0.05; **p < 0.01; ***p < 0.001 compared to indicated group/control.

A number of potential mechanisms could account for the reduced functional responses of the P2X7R in the contralateral hippocampus. An effect of reduced transcription was ruled out because *P2rx7* mRNA levels showed minimal changes in the contralateral hippocampus after status epilepticus (Fig. 2A). We therefore explored the possibility that contralateral P2X7R function differed because of reduced protein levels. Western blotting determined that P2X7R protein levels were reduced in the contralateral hippocampus following status epilepticus (Fig. 2B). P2X7R immunoreactivity was also reduced in the contralateral hippocampus, particularly in the stratum lucidum of the contralateral CA3 subfield representing the terminal fields of the mossy fibers from dentate granule cells (Fig. 2C). Levels of P2X7R protein were also reduced in the synaptodendritic compartment on the contralateral side (Fig. 2D). To support these findings, we assessed levels of IL-1β which lies downstream of P2X7R activation^12^,^41^. Consistent with reduced P2X7R function, contralateral levels of IL-1β were lower relative to the ipsilateral hippocampus after status epilepticus (Fig. 2E).

We next investigated post-transcriptional targeting of P2X7R by miRNA^21^,^22^ as the potential mechanism responsible for reduced protein levels. Proper miRNA-directed targeting requires binding of mRNA transcripts to Ago2 within the RISC^25^,^42^. To determine if *P2rx7* mRNA was targeted to the RISC we immunoprecipitated Ago2 from mouse hippocampus and measured levels of *P2rx7* by quantitative PCR. Increased RISC-loaded *P2rx7* mRNA was detected in the contralateral but not ipsilateral hippocampus, after status epilepticus (Fig. 2F).

To identify potential miRNAs targeting the *P2rx7* transcript in the contralateral hippocampus we analyzed RISC-bound miRNA (Fig. 2G). This approach ensures highest predictive value for identifying functional miRNAs^42^. Following Ago2 pull-downs, a high-throughput OpenArray (OA) platform was used to perform genome-wide screening of mouse miRNAs. After profiling (*see* Supplementary data file), we separated those miRNA that were eluted from the contralateral hippocampus after status epilepticus from miRNAs present in the RISC in the ipsilateral hippocampus after status epilepticus and non-seizure controls. This resulted in a set of 24 miRNAs unique to the RISC in the contralateral hippocampus (Fig. 2G; Note: one of these, miR-1186, was eliminated as it is no longer considered a *bone fide* miRNA^39^). The most highly expressed miRNA unique to the contralateral hippocampus was miR-22-3p (hereafter miR-22) (Fig. 2G). The mature sequence of miR-22 is fully conserved between mouse and human (Fig. 2H). We then validated the OA profiling result by individual Taqman miRNA assay and this confirmed miR-22 levels were increased in Ago2-eluted samples exclusively on the contralateral side after status epilepticus (Fig. 2I). Two other brain-expressed miRNAs that could potentially target *P2rx7* (miR-186 and miR-150)^21^, were not uploaded to the RISC in the contralateral hippocampus (Fig. 2J).

*In silico* analysis identified a putative miR-22 seed binding site in the 3′UTR of the *P2rx7* transcript, comprising an 8 nt match, starting at the second nt (adenosine) from the 5′ end of the miRNA (Supplementary Fig. 2A). To support functional targeting of *P2rx7* mRNA by miR-22, we transfected neuronal cells (N2a) with either LNA-modified antagomirs to inhibit miR-22 (Ant22) or a mimic sequence (Mi22) to upregulate miR-22, and then recorded P2X7R agonist-evoked currents (Fig. 2K). Ant22 treatment resulted in potentiation of BzATP-evoked currents whereas cells transfected with Mi22 displayed reduced BzATP-evoked currents (Fig. 2K).

We next explored which cells expressed miR-22 in mouse brain tissue sections. *In situ* hybridization detected a strong miR-22 signal in granule layer cells as well as hilar and pyramidal neurons and smaller cells, likely glia, as well as in the neocortex and other brain regions (Fig. 2L and Supplementary Fig. S2B). The distribution of miR-22 was similar in tissue sections from mice after status epilepticus (Fig. 2L). Again, a strong signal was detected in granule layer cells and glia-like cells in both the ipsilateral and contralateral hippocampus (Fig. 2L). This pattern of cellular expression was confirmed in purified cultures of mouse hippocampal neurons, microglia and astrocytes, with highest basal levels of miR-22 found in cultured astrocytes (Fig. 2M).

### Inhibition of miR-22 increases P2X7R protein and function *in vivo*

**Figure 3:**
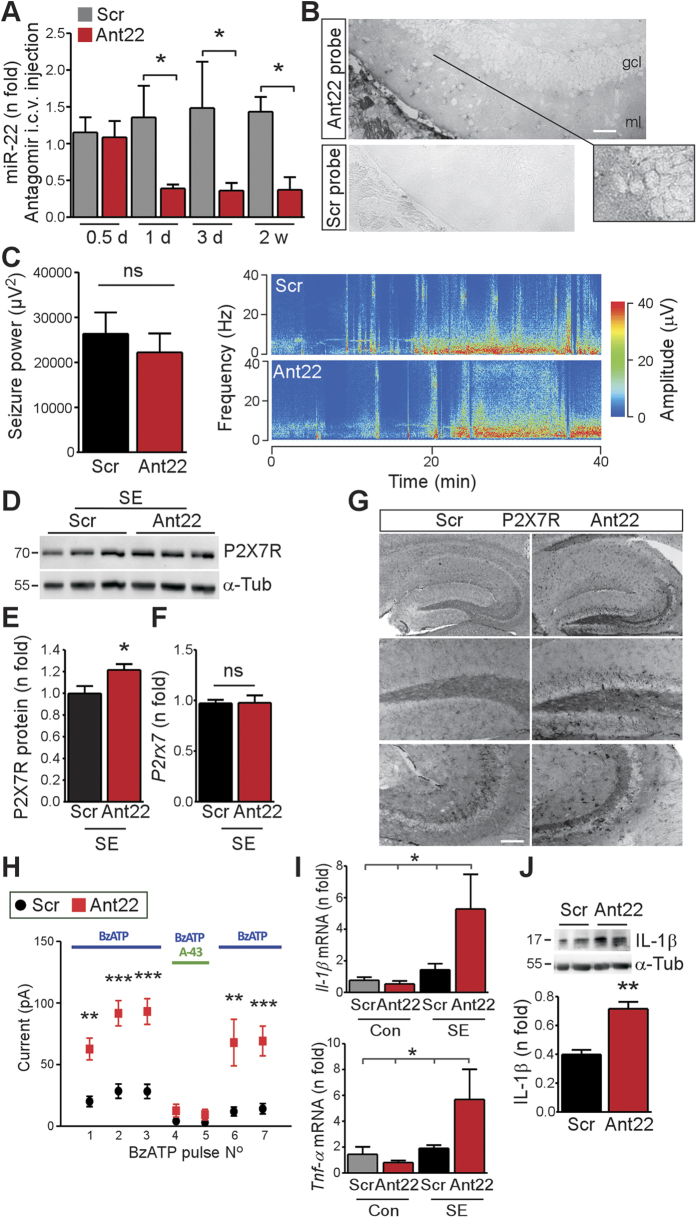
miR-22 manipulation alters P2X7R function *in vitro* and *in vivo*. (**A**) Graph showing temporal course of miR-22 down-regulation in hippocampal tissue after Ant22 i.c.v. injection (d, day; w, week; *n* = 4/group). (**B**) *In situ* hybridization for A*n*t22 in the hippocampus 24 h after Ant22 injection. Scale bar, 50 μm. Inset on right shows a high magnification view of a group of cells. Panel below shows absence of staining in an Ant22 mouse section stained for the scrambled probe. (**C**) EEG data showing seizure power (*n* = 5–6/group) and representative EEG heat-map for Ant22 and Scr mice during SE. (**D,E**) Immunoblot (*n* = 1/lane) and graph showing increased P2X7R protein levels in the contralateral hippocampus 12 h after SE in Ant22 treated mice when compared to controls (*n* = 4/group). (**F**) No difference in *P2rx7* mRNA between Scr and Ant22 in the contralateral hippocampus 12 h after SE (*n* = 4/group). (**G**) Photomicrographs showing increased P2X7R immunoreactivity in Ant22 mice compared to Scr in contralateral hippocampal subfields 12 h after SE. Scale bar, 100 μm. (**H**) Patch-clamp recordings from EGFP-positive dentate granule cells from the contralateral hippocampus 12 h after SE showing increased BzATP-evoked currents in Ant22-treated mice when compared to Scr mice (*n* = 9 (Ant22) and 8 (Scr) cells from 4 (Ant22) and 5 (Scr) mice). (**I**) Graphs show transcript levels of proinflammatory cytokines are elevated in the contralateral hippocampus of Ant22 treated mice at 12 h after SE (*n* = 5 – 6/group). (**J**) Western blot (*n* = 1/la*n*e) and graph show increased IL-1β i*n* the contralateral hippocampus of Ant22 mice 12 h after SE (*n* = 4/group). *p < 0.05; **p < 0.01 compared to indicated group/control.

To obtain *in vivo* evidence that P2X7R is a target of miR-22 we tested the effects of inhibiting miR-22 in mice using antagomirs. Microinjection of 0.5 nmol Ant22 into the lateral ventricle of naïve mice resulted in a 65–70% reduction in miR-22 levels measured 24 h later compared to a scrambled sequence (Scr), and this knockdown persisted for at least two weeks (Fig. 3A and Supplementary Fig. S3). Ant22 did not alter levels of two other tested miRNAs (Supplementary Fig. S3). *In situ* hybridization performed on sections 24 h after antagomir injection in naïve mice confirmed Ant22 transfected into hippocampal cells (Fig. 3B).

We proceeded next to study the effect of silencing miR-22 on seizures. Antagomir-mediated silencing of miRNA takes up to 12 h to produce significant knock-down and is maximal 24 h following i.c.v. injection^27^. We therefore injected Ant22 or Scr into the mouse ventricle and then 24 h later animals were subject to status epilepticus. EEG recordings determined that Ant22-injected mice displayed similar overall seizure severity during status epilepticus compared to Scr animals (Fig. 3C). We next investigated molecular changes 12 h later, finding P2X7R protein but not transcript levels were by then higher in the contralateral hippocampus of Ant22-treated mice compared to Scr animals (Fig. 3D–F). Increased P2X7R immunoreactivity was also apparent in the contralateral hippocampus of Ant22-injected mice after status epilepticus (Fig. 3G). P2X7R protein levels were not significantly increased in the ipsilateral hippocampus from Ant22-treated mice compared to Scr samples (Supplementary Fig. S4).

To investigate whether miR-22 inhibition also affected function of the P2X7R, we performed patch-clamp recordings from EGFP-positive dentate granule cells in *ex vivo* slices obtained from Scr- and Ant22-treated *P2rx7* reporter mice 12 h after status epilepticus (Fig. 3H). BzATP-evoked currents were higher in the contralateral hippocampus of Ant22 mice subjected to status epilepticus compared to post-status animals given Scr (Fig. 3H). Confirming this increase was P2X7R-specific, evoked currents were reversibly abolished by application of a specific P2X7R antagonist (Fig. 3H). Taken together, these findings are consistent with miR-22 targeting of P2X7R after status epilepticus in the contralateral hippocampus.

### Silencing miR-22 increases markers of inflammation in the contralateral hippocampus

An increase in P2X7R function would be expected to promote neuroinflammation via IL-1β. We therefore assessed P2X7R-associated neuroinflammatory markers after status epilepticus in mice treated with Ant22 or the scrambled sequence. Levels of IL-1β and another key inflammatory cytokine, TNFα, were similar to control (non-status epilepticus) mice in the contralateral hippocampus of Scr animals 12 h after status epilepticus (Fig. 3I). In contrast, levels of IL-1β (transcript and protein) and *Tnfα* were elevated at this time point after status epilepticus in the contralateral hippocampus of Ant22-treated mice (Fig. 3I,J). This suggests de-repression of P2X7R by silencing miR-22 generates an early pro-inflammatory environment in the post-status contralateral hippocampus. IL-1β levels were not significantly increased in the ipsilateral hippocampus from Ant22-treated mice compared to Scr samples at this time point (Supplementary Fig. S4). Inflammation markers *IL-1β* and *Tnfα* were also significantly elevated at 72 h after status epilepticus in the contralateral hippocampus of Ant22 mice compared to Scr mice (Supplementary Fig. S5).

### *In vivo* silencing of miR-22 exacerbates epilepsy

**Figure 4:**
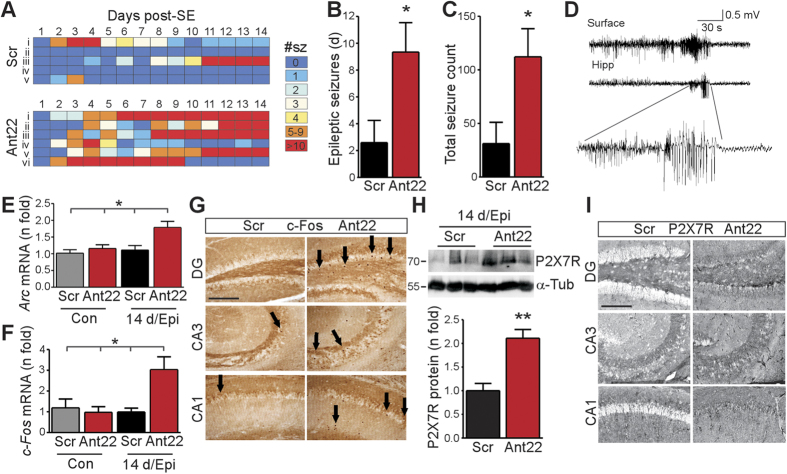
miR-22 inhibition exacerbates epilepsy in mice. (**A–C**) Individual mouse (i–v/vi) daily seizure counts and summative graphs showing spontaneous seizures during 14 days recording in mice treated with Ant22 or Scr before SE (*n* = 5–6/group). (**D**) Representative surface and contralateral hippocampal depth electrode recording of a spontaneous seizure on day 4 after SE in an Ant22 mouse. (**E,F**) Increased *Arc* and *c-Fos* in the contralateral hippocampus of epileptic Ant22 mice at the end of recordings compared to epileptic Scr mice and non-kainic acid controls (*n* = 11 (Ant22 Cont), 12 (Scr Cont), 16 (Ant22 SE) 13 (Scr SE). (**G**) Immunostaining for c-Fos shows more positive cells (arrows) in epileptic Ant22-treated mice in the contralateral hippocampus compared to Scr-treated mice at the end of epilepsy monitoring. Scale bar = 200 μm. (**H**) Immunoblot (*n* = 1/lane) and corresponding graph showing increased P2X7R levels in the contralateral hippocampus of epileptic Ant22-treated mice when compared to epileptic Scr mice at the end of epilepsy monitoring (*n* = 4–5/group). (**I**) Increased P2X7R immunoreactivity in epileptic A*n*t22-treated mice compared to epileptic Scr-treated mice in all contralateral hippocampal subfields (DG, CA3 and CA1). Scale bar = 200 μm. *p < 0.05; **p < 0.01 compared to indicated group/control.

Based on these neuroinflammatory responses, we hypothesized that miR-22 inhibited mice would develop exacerbated epilepsy. To test this idea, we injected mice with either Ant22 or Scr, subjected them to status epilepticus 24 h later, and then performed two weeks of continuous EEG-telemetry recordings^27^. The first spontaneous seizures in Scr mice were detected 2–4 days after status epilepticus and their frequency averaged ~3 per day over the course of recordings, consistent with previous reports in this model^27^ (Fig. 4A,B). Ant22 mice also displayed first spontaneous seizures 2–4 days after status epilepticus but the frequency of spontaneous seizures in Ant22 mice rapidly accelerated over time. By day 5, half of the Ant22 mice were having 5 or more seizures per day and seizure frequency over the two weeks was approximately four times higher in Ant22 mice compared to Scr mice (Fig. 4A–C). Intrahippocampal EEG recordings in Ant22 mice during the first week post-status also detected spontaneous seizures involving the contralateral hippocampus (Fig. 4D).

After epilepsy monitoring, we analyzed contralateral hippocampal samples for molecular markers of seizure activity. In epileptic Scr mice, levels of both c-Fos and Arc were found to be similar to non-status epilepticus controls (Fig. 4E–G), consistent with minimal recruitment of the contralateral hippocampus. In contrast, levels of c-Fos and *Arc* were both elevated in the contralateral hippocampus of Ant22-treated epileptic mice, consistent with spontaneous seizures affecting the contralateral hippocampus (Fig. 4E–G). P2X7R protein levels were also higher in the contralateral hippocampus of epileptic Ant22 mice at the end of recordings (Fig. 4H,I). In the ipsilateral hippocampus of epileptic mice, levels of P2X7R were similar between Ant22 and Scr mice (Supplementary Fig. S6). Ipsilateral levels of *c-Fos* were also similar between the groups while *Arc* expression was slightly increased in Ant22 mice (Supplementary Fig. S6). Thus, silencing miR-22 exacerbates epilepsy and leads to contralateral hippocampal involvement in spontaneous seizures.

### *In vivo* silencing of miR-22 exacerbates astrogliosis

**Figure 5:**
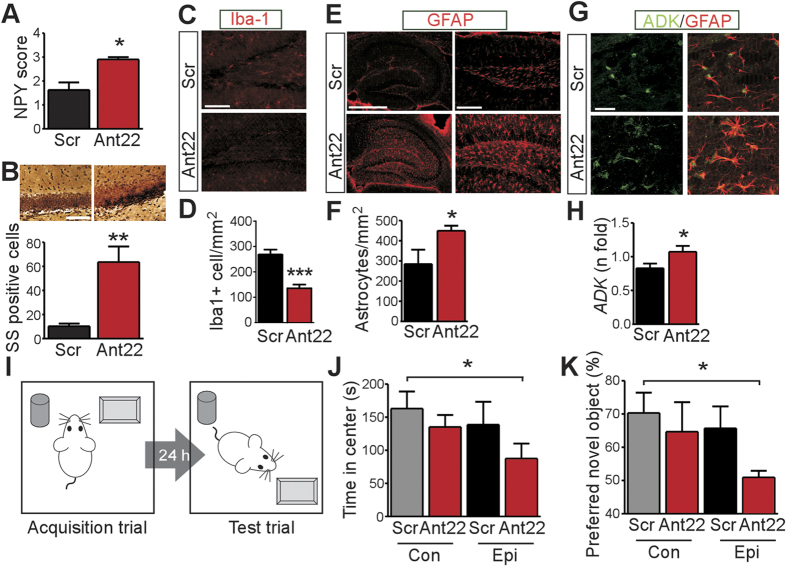
miR-22 inhibition increases astrogliosis and impairs cognitive performance in epileptic mice. (**A,B**) Epileptic Ant22 mice at the end of epilepsy monitoring display (*A*) increased neuropeptide Y (NPY) scores, a marker of synaptic rearrangement (*n* = 5–6/group) and (*B*) increased silver staining (SS) of reversibly damaged cells (*n* = 9–10/group). (**C,D**) Epileptic Ant22 mice display reduced staining for microglia in the contralateral hippocampus (*n* = 10–11/group). (**E–H**) Epileptic Ant22 mice display increased numbers of GFAP positive astrocytes and increased ADK (adenosine kinase) protein and immunostaining (*n* = 13–14/group). Scale bar in *C*, 150 μm; *E*, left; 1 mm; right, 200 μm. *G*, 20 μm. (**I**) Cartoon showing behavioral testing paradigm. Mice were presented with two objects and then, after a one day delay, re-presented but with one object moved (original drawing by author DC Henshall). (**J,K**) Behavioral tests on control and epileptic Ant22 and Scr mice at the end of recordings. Graphs show (**J**) percentage of time spent in the central area during habituation period of object location memory task. Epileptic Ant22 mice spent less time in the center area suggesting increased anxiety (*n* = 11–14/group). (**K**) Graph showing the percentage preference for the displaced object. Epileptic Ant22 mice showed reduced preference for novel object location (*n* = 8–12/group). *p < 0.05; **p < 0.01; ***p < 0.001 compared to control or indicated group.

We next examined pathological changes in the hippocampus after epilepsy monitoring in Ant22 and Scr mice. Tissue sections were stained for neuropeptide Y, a marker of mossy fibers to assess synaptic reorganization^27^ and silver staining for potential neuronal injury. The contralateral hippocampus from epileptic Ant22 mice displayed increased markers of both synaptic reorganization and neuronal injury, compared to Scr mice (Fig. 5A,B). We next stained sections for Iba1, a marker of microglia, since P2X7R activation promotes microglial proliferation and activation^13^. Unexpectedly, microglia counts were lower in the contralateral hippocampus from epileptic Ant22 mice compared to Scr mice (Fig. 5C,D). We then investigated astrogliosis, another conserved pathologic feature of focal epilepsies. Astrogliosis may also be triggered indirectly via P2X7R-mediated release of IL-1β^41^. Astrocyte staining and astrocyte counts were increased in the contralateral hippocampus of epileptic Ant22 mice compared to Scr animals (Fig. 5E,F). Molecular markers of astrogliosis were also elevated in the contralateral hippocampus of Ant22 mice, including glial fibrillary acidic protein (GFAP) and adenosine kinase (Fig. 5E,G,H). Microglia and astrocyte counts in the ipsilateral hippocampus were not significantly different between Ant22 mice and Scr (Supplementary Fig. S7).

To determine if these cellular changes and the exacerbated epilepsy had effects on animal behavior we tested epileptic Ant22 and Scr mice using a novel object relocation test (Fig. 5I)^34^. Mice were introduced to an arena to explore two objects. One day later they were re-introduced to the test area with one object displaced to a new position; animals with normal hippocampal function spend more time exploring the moved object^40^. The test also enables an analysis of anxiety during the exploratory phase, based on time spent in the central area compared to the periphery. Epileptic Ant22 mice spent less time in open areas during monitoring compared to Scr-treated epileptic mice and non-epileptic control animals, suggesting increased anxiety (Fig. 5J). Epileptic Ant22 mice also failed to show a preference in the novel object re-location test (Fig. 5K), suggesting impaired hippocampal function.

### Pro-inflammatory phenotype in miR-22-silenced mice is P2X7R-dependent

**Figure 6:**
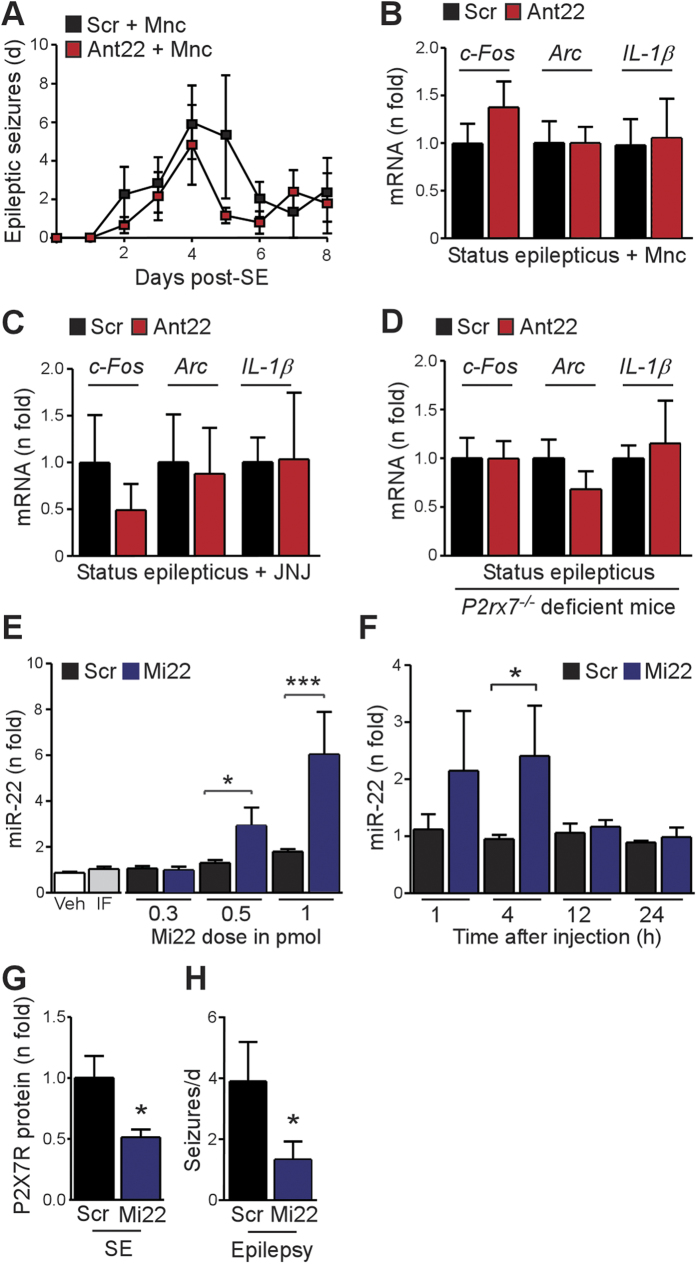
Involvement of P2X7R in the mechanism of Ant22-mediated exacerbation of epileptic phenotype and *in vivo* anti-seizure effect of Mi22. (**A**) Graph showing treatment of Ant22 mice with the broad spectrum anti-inflammatory drug minocycline (Mnc; twice daily, (30 mg/kg) beginning 1 h post SE) normalized the epileptic phenotype to the Scr level during continuous telemetric EEG recordings (*n* = 4/group). (**B**) Mnc treatment normalized expression of neuronal activity genes and neuroinflammtory markers in the contralateral hippocampus of Ant22 mice (*n* = 4/group). (**C**) Treatment of mice with the specific P2X7R inhibitor JNJ-47965567 (JNJ, twice daily (30 mg/kg) for 3 consecutive days starting at 1 h post SE) also normalized expression of *c-Fos*, *Arc*, and *Il-1β* in the contralateral hippocampus at 72 h post-status epilepticus (*n* = 5/group). (**D**) Graph showing Ant22-treated *P2rx7*^*−/−*^ mice display scramble-injected levels of *c-Fos*, *Arc* and *Il-1*β in the contralateral hippocampus at 72 h post-status epilepticus (*n* = 5/group). (**E**) Dose-range study showing hippocampal levels of miR-22 measured 4 h after i.c.v. injection of mimic (Mi22) at picomolar doses. Note that 0.5 picomolar produces a ~3 fold increase closely matching the increase found in the contralateral hippocampus after SE (*n* = 11 (Veh), 10 (Mi22 0.5 pmol), 8 (Mi22 0.3 pmol) and 5 (IF (infectamine-only), Scr 0.3 pmol and Scr andMi22 at 1 pmol)). (**F**) Time course showing injection of 0.5 pmol Mi22 results in a transient increase in miR-22 levels in the hippocampus (*n* = 4–7/group). (**G**) Graph showing decreased P2X7R protein levels in the ipsilateral hippocampus 8 h after SE in mice injected with 0.5 pmol Mi22 given 1 h after status epilepticus (*n* = 4–5/group). (**H**) Mi22 treated mice (Mi22; 0.5 pmol injected at 1, 8 and 24 h post-SE) show reduced spontaneous seizures during the first 4 days after status epilepticus (*n* = 7/group). *p < 0.05; **p < 0.01; ***p < 0.001 compared to indicated group/control.

To determine if the exacerbated epilepsy phenotype in Ant22-treated mice was related to neuroinflammation, we treated mice with the broad-spectrum anti-inflammatory drug minocycline. Scr or Ant22 mice received twice daily injections of minocycline and spontaneous seizure frequency was recorded for a week after status epilepticus. Minocycline normalized the spontaneous seizure rate in Ant22 mice (Fig. 6A) (seizure counts: Scr, 22 ± 9; Ant22, 16 ± 5; P = 0.54). Minocycline treatment also normalized contralateral levels of molecular markers of seizure activity and neuroinflammation in Ant22 mice (Fig. 6B).

To specifically link the neuroinflammatory effects of Ant22 to increased P2X7R function we treated additional Scr or Ant22 mice with JNJ-47965567, a potent and selective inhibitor of P2X7R^43^. Unlike A-438079 (used in the cell studies above), JNJ-47965567 is centrally available for several hours following systemic administration in rodents^43^. Treatment of Ant22 mice with twice daily injections of JNJ-47965567 normalized levels of molecular markers of increased neuronal activity and inflammation in the contralateral hippocampus to levels in Scr-treated mice (Fig. 6C). To complement these pharmacologic data we obtained genetic evidence for P2X7R involvement in the Ant22 mouse phenotype, investigating the inflammatory and hyperexcitability markers in *P2rx7*-deficient mice. Ant22-mediated increases in contralateral hippocampal levels of *c-Fos*, *Arc* and *IL-1β* expression were all blocked in *P2rx7*^*−/−*^ mice subjected to status epilepticus (Fig. 6D) confirming the pro-excitability and pro-inflammatory effects of Ant22 were mediated by the P2X7R.

### miR-22 mimic treatment reduces P2X7R expression and seizures *in vivo*

Last, we sought to complement the antagomir findings and explore therapeutic potential by investigating whether over-expression of miR-22 would suppress P2X7R protein levels in the contralateral hippocampus and reduce spontaneous seizures. Mimics targeting miR-34a have recently entered clinical trials for the treatment of cancer^44^ and we therefore attempted to directly upregulate miR-22 in the hippocampus by intracerebroventricular microinjection of Mi22. Injection of picomolar amounts of Mi22 produced dose-dependent, short-lasting increases in hippocampal miR-22 levels (Fig. 6E,F). Levels of two other miRNAs, miR-19a and miR-92, were not changed by Mi22 (data not shown). Injection of 0.5 pmol Mi22 most closely matched the fold increase in the contralateral hippocampus of mice after status epilepticus and was used for the next experiments. Western blotting determined that P2X7R protein levels were lower in mice injected with Mi22 after status epilepticus (Fig. 6G). Finally, we analyzed spontaneous seizure rates in Mi22-treated mice. Injection of Mi22 after status epilepticus reduced the occurrence of spontaneous seizures during the first 4 days of epilepsy monitoring (Fig. 6H). However, seizure rates during later monitoring (days 5–9) were similar to Scr levels (data not shown), perhaps as a result of the transient ability of Mi22 to enhance hippocampal miR-22 levels (*see* Fig. 6F).

## Discussion

The present study supports a role for post-transcriptional control of the P2X7R in epilepsy. We found that miR-22 and the *P2rx7* transcript were selectively uploaded to the RISC in the normally spared contralateral hippocampus of mice after status epilepticus triggered by intra-amygdala kainic acid. De-repression of the miRNA target by antagomirs triggered inflammatory responses that were not ordinarily seen in this brain region, and increased spontaneous seizures in mice, whereas delivery of miR-22 suppressed seizures. Together, these data reveal a novel pathway functioning to suppress inflammation via the P2X7R that may represent a therapeutic target in epilepsy or diseases of neuroinflammation.

The importance of neuroinflammation in epileptogenesis and maintenance of the epileptic state continues to be debated but has nevertheless prompted clinical trials of anti-inflammatory drugs for epilepsy^2^,^45^. Identification of additional cell and molecular mechanisms controlling neuroinflammation may yield further opportunities for seizure control and anti-epileptogenesis. Findings here point to the P2X7R as an important target in this regard. Although protective roles are known^46^, activation of the P2X7R has pro-inflammatory and pro-convulsive effects, foremost by triggering IL-1β release from cells^12^,^41^. Released IL-1β rapidly enhances glutamergic (AMPA) signaling in neurons and promotes astrogliosis^41^. P2X7R activation can also directly modulate hippocampal neurotransmitter release^14^,^15^. The major finding in the present study was that expression of the P2X7R is regulated post-transcriptionally by miR-22 within the contralateral hippocampus after status epilepticus and this restrains the emergent epilepsy phenotype. This extends work in non-CNS models that showed targeting of the P2X7R by miRNA^21^,^22^ and provides functional evidence for post-transcriptional control of gene expression by miRNAs in epilepsy, which remains limited^26^.

The present study used two strategies to identify miRNAs that have not previously been undertaken in profiling studies of status epilepticus^47^. First, we took advantage of the unique asymmetric feature of the intra-amygdala kainic acid model, focusing on the contralateral hippocampus. Second, our study took account of evidence that analysis of RISC-loaded miRNA improves the accuracy of predicting miRNA function^28^,^42^. Notably, the miR-22 *in situ* signal was readily observed in ipsilateral neurons including dentate granule cells, where there was no RISC loading of the *P2rx7* transcript. This suggests co-localisation of miRNA and target in the same cell cannot be taken as evidence of functional targeting and emphasizes the importance of Ago2 analysis. Combining both approaches identified miR-22 as the most abundant RISC-loaded miRNA unique to the contralateral hippocampus after status epilepticus. This miRNA has not been linked to epilepsy in the majority of experimental and human studies, including work from our own group^26^,^48^,^49^,^50^,^51^. It may have been missed because profiling did not feature RISC analysis or because studies focused only on typical sites of damage (e.g. ipsilateral hippocampus). In animal studies where altered miR-22 expression was reported, the direction of change after status epilepticus was inconsistent^52^,^53^. Since the main strategy to find epilepsy-associated miRNAs has been to profile total miRNA in damaged brain regions after status epilepticus^47^, our study offers both a novel approach that enriches for potentially protective miRNAs and technical advance over previous work that profiled miRNA without consideration for functionality.

Early work reported tumor-suppressor effects of miR-22^54^ but anti-inflammatory and neuroprotective functions recently emerged for miR-22 in CNS models^55^,^56^. Consistent with a role in suppressing neuroinflammation, we found that blocking miR-22 resulted in an early and sustained elevation in proinflammatory signaling and molecular markers of excitability in the contralateral hippocampus. Silencing miR-22 also caused a rapid escalation of epileptic seizure rates in mice. These findings suggest miR-22 normally restrains development of a contralateral epileptogenic focus in this model and loss of miR-22, as with loss of miR-128^28^, is pro-epileptic. Determining which of the multiple potentially pro-epileptogenic effects of silencing miR-22 is most critical for the epilepsy phenotype will require further studies. Understanding what controls miR-22 activation may help explain why secondary epileptic foci form in some cases. Answers to both have clinical relevance because surgical resection of the primary seizure focus in TLE patients does not always relieve intractable epilepsy. The role of contralateral brain structures is important and molecular markers that predict whether or not these foci exist would be valuable^5^,^57^,^58^.

Among several miRNAs that could potentially target P2X7R, only miR-22 was up-loaded into the RISC. This agrees with the recognized specificity of RISC loading and miRNA-target selection, although the guiding mechanisms are not fully understood^25^,^42^. We did not observe a reduction in *P2rx7* transcript levels due to miR-22 targeting. This may be because of concurrent transcription or the miRNA working via translational repression^59^. While miRNA-mediated targeting is expected to lead to degradation of the target mRNA, our findings are consistent with other studies that did not find mRNA reductions after miRNA targeting in neurons *in vitro*^30^ and in the hippocampus *in vivo*^60^. Our study adds to the known targets of miR-22 and to the miRNAs for which ion channels have been identified as targets in the brain^54^,^55^,^56^,^61^.

Brain-enriched miRNAs can have over one hundred mRNA targets^28^. This multi-targeting action is potentially attractive for anti-epileptogenesis because it may be necessary to block several signaling components in the same or interacting pathways to achieve disease-modifying effects. However, data here show that blocking P2X7R using genetic or pharmacologic approaches was sufficient to obviate the main pro-inflammatory and pro-excitatory phenotype in miR-22-inhibited mice. This suggests that, despite multi-targeting potential of miRNAs, the main effects of miR-22 in the contralateral hippocampus are via P2X7R. We note as well that P2X7R activation can directly result in increased cellular levels of c-Fos^62^, and this could contribute pro-epileptic effects^63^. The observed increases in c-Fos in Ant22 mice might therefore be a combination of seizure activity and direct effects of P2X7R function. We also found that miR-22 modulation affected *Tnfα* levels. This may be downstream of P2X7R-mediated NfκB activation^64^ and is a secondary readout of P2X7R function and neuroinflammation. In contrast, there have been reports of anti-convulsant effects of the P2X7R^65^. Although this may be specific to inflammatory conditions in certain models, it is consistent with recent evidence that the timing of intervention in ATP-mediated inflammatory signaling is critical and that premature intervention could have deleterious effects on brain injury^46^. Thus, while our data point to miR-22 protecting via suppression of P2X7R and thus an anti-inflammatory mechanism, inflammatory signaling is not invariably pro-epileptogenic.

An interesting observation in the present study was the dramatic astrogliosis found in the contralateral hippocampus of miR-22 silenced epileptic mice. This is a plausible cause of the increased spontaneous seizures^66^,^67^,^68^. The astrogliosis is likely a non-cell autonomous effect of Ant22 because the *P2rx7* transcript is not expressed in astrocytes in the present model (present data and^11^,^17^,^18^). Thus, the astrogliosis in Ant22 mice may be due to de-repression of P2X7R in other cells, likely neurons or microglia, which then drives astrocytosis via IL-1β and further release of neurotoxic and pro-convulsive mediators^41^,^69^. Other miRNAs have been reported to prevent astrogliosis, including miR-146a^29^, although *in vivo* functional data on this miRNA have not been presented for epilepsy. An unexpected finding was that microglia counts were lower in the contralateral hippocampus of epileptic Ant22 mice despite the known role of P2X7R in microglia proliferation^13^. High P2X7R levels, however, render microglia susceptible to ATP-induced apoptosis^70^ so it is possible silencing miR-22 promoted microglial cell death. Although microglia are the likely source of IL-1β and perhaps other pro-convulsive molecules, their loss could exert pro-convulsive effects because microglia modulate synaptic structure and function^71^. Further studies will be required to determine which cellular effect of miR-22 silencing is the most important contributor to the epilepsy phenotype.

It will require additional studies to determine why the seizure “dose” that reaches the contralateral hippocampus induces the pathway and why induction of miR-22 is not effective in the ipsilateral hippocampus. This may be due to the Ago2 search and selection process^72^ or the transcriptional control of miR-22, the mechanisms of which are unknown in the brain. Notably, specificity protein 1 (Sp1) was recently identified to control expression of P2X7R in neurons^73^ and the miR-22 promoter contains putative Sp1 binding sites (C.C. personal communication). Dual control of miRNA and target by the same transcription factor fits well with the emerging understanding of the role of miRNAs in fine-tuning protein levels and maintaining gene expression patterns in response to cell stress^74^.

Finally, the present study demonstrates an *in vivo* anti-seizure effect of a miRNA mimic. RNA therapeutics including miRNA mimics have recently entered clinical testing in humans^44^ and we found that delivery of a sub-picomolar central injection of Mi22 was sufficient to upregulate miR-22 to a level comparable to that in the contralateral hippocampus and this had an anti-seizure effect. Mimic effects on miR-22 and seizures were short-lived, however, contrasting the prolonged effects of antagomirs in this study and reported previously^27^. Advances in methods for the sustained release of miRNA at safe therapeutic doses^75^ may facilitate deployment of RNA therapeutics to augment miRNA levels for epilepsy and other neurological disorders.

## Additional Information

**How to cite this article**: Jimenez-Mateos, E. M. *et al.* microRNA targeting of the P2X7 purinoceptor opposes a contralateral epileptogenic focus in the hippocampus. *Sci. Rep.* **5**, 17486; doi: 10.1038/srep17486 (2015).

## Supplementary Material

Supplementary Information

Supplementary Data set
