# Supplementary material for: microRNA targeting of the P2X7 purinoceptor opposes a contralateral epileptogenic focus in the hippocampus
Source: Sci Rep. 2015 Dec 3;5:17486. doi: 10.1038/srep17486 (PMC4668358; doi:10.1038/srep17486)
Supplement: Supplementary Information [file srep17486-s1.pdf]

Supplementary Figures S1 – S7

microRNA targeting of the P2X7 purinoceptor opposes a contralateral epileptogenic focus in the hippocampus

Eva M. Jimenez-Mateos, Marina Arribas-Blazquez, Amaya Sanz-Rodriguez, Caoimhin Concannon, Luis A. Olivos-Ore, Cristina R. Reshke, Claire M. Mooney, Catherine Mooney, Eleonora Lugara, James Morgan, Elena Langa, Alba Jimenez-Pacheco, Luiz Fernando Almeida Silva, Guillaume Mesuret, Detlev Bosion, M. Teresa Miras-Portugal, Michael Letavic, Antonio R. Artalejo, Anindya Bhattacharya, Miguel Diaz Hernandez, David C. Henshall, Tobias Engel

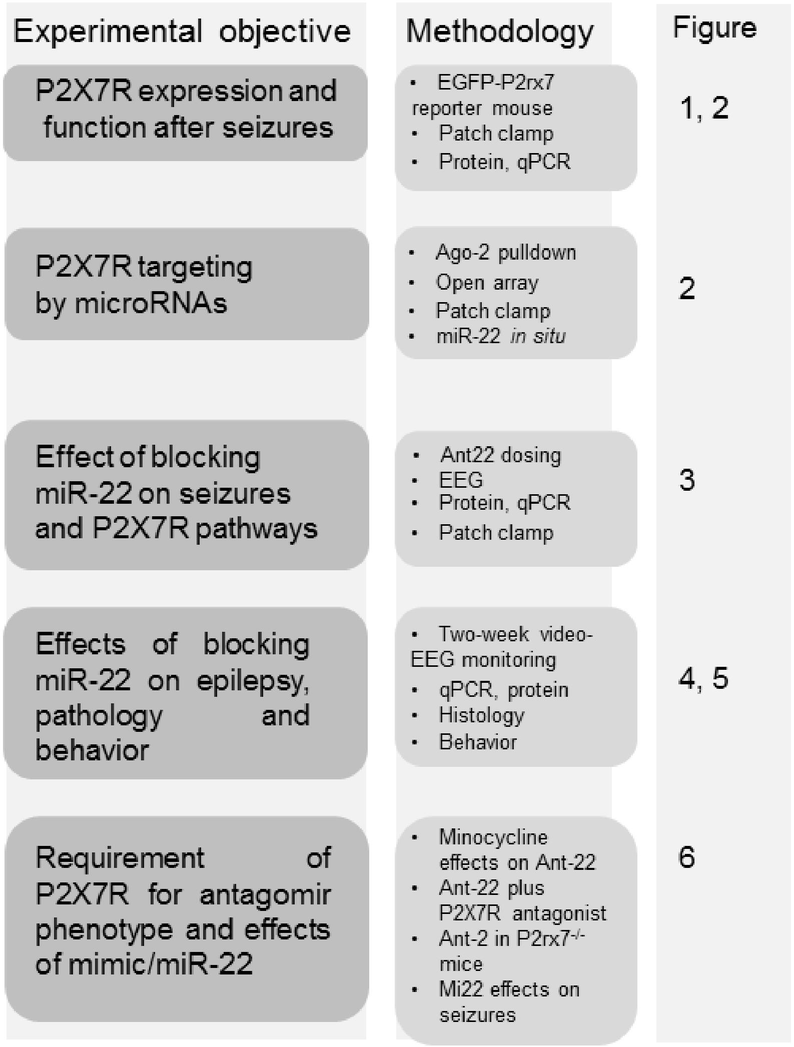

Supplementary data Figure S1. A flow chart showing an overview of the experiments.

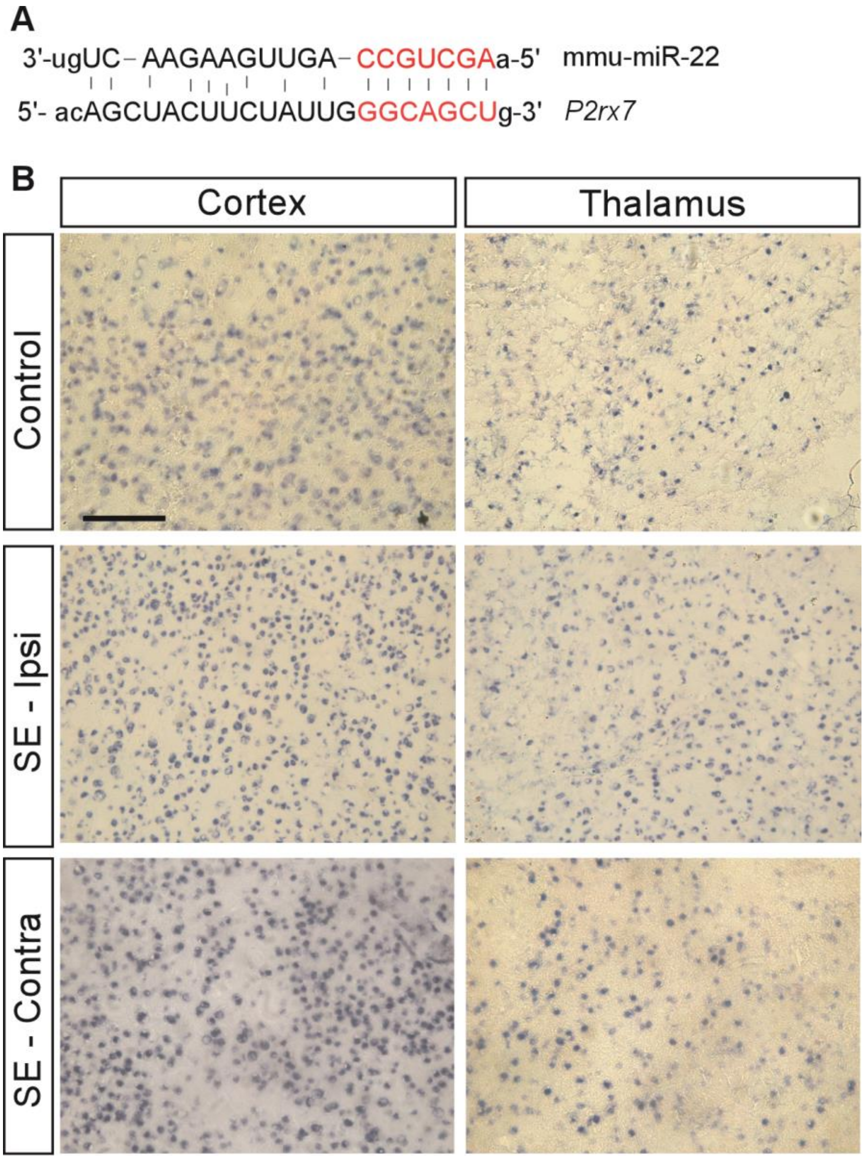

Supplementary data Figure S2. *Sequence alignment of miR-22 and in situ hybridization for miR-22 in extra-hippocampal brain regions.* (A) Theoretical sequence alignment of mouse miR-22 with *P2rx7* (based on predictions made using algorithm in microrna.org). Area in red shows predicted seed region. Vertical dashes indicate potential base pairing. (B) Images show representative miR-22 *in situ* in the cortex and thalamus for both control and post-SE brain regions (8 h). Scale bar, 150 µm

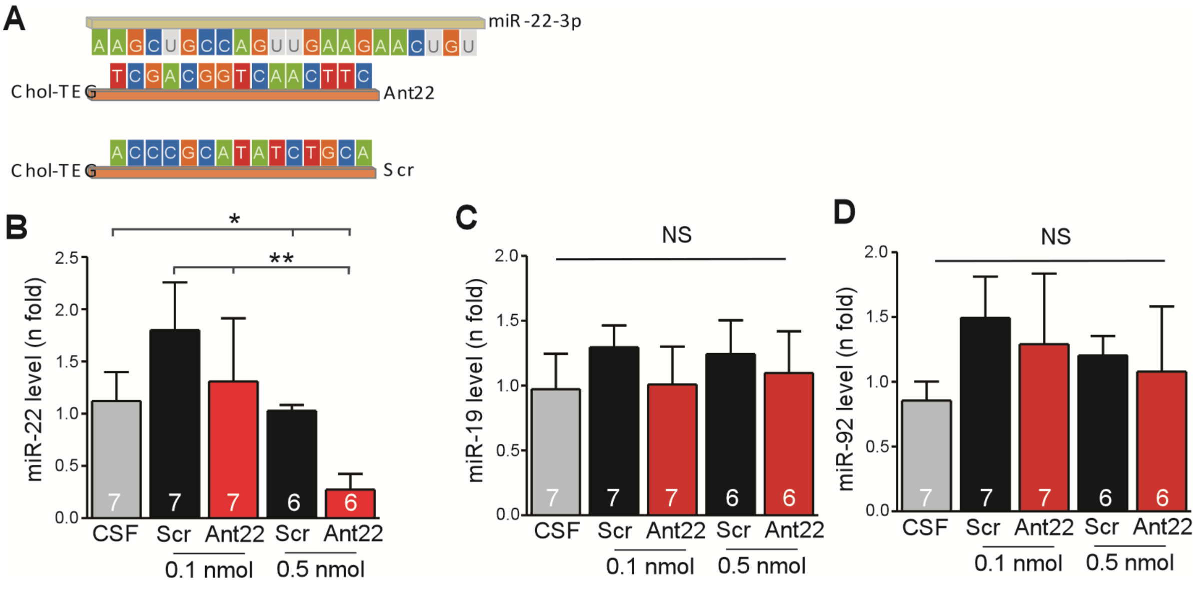

Supplementary data Figure S3. *Specificity of Ant22*. **(A)** Antagomir and scrambled sequence. **(B)** Dose-range finding study with antagomirs against miR-22. At 0.1 nmol, Ant22 did not significantly reduce miR-22 levels in the hippocampus compared to a scrambled version (Scr) or cerebrospinal fluid (CSF) measured 24 h after i.c.v. injection, whereas 0.5 nmol i.c.v. injection significantly reduced miR-22 hippocampal levels. **(C, D)** Graphs showing effect of 0.1 and 0.5 nmol Ant22 on levels of two other miRNAs in the hippocampus at 24 h. Group sizes indicated on individual bars. *$p < 0.05$; ** $p < 0.01$ compared to indicated group.

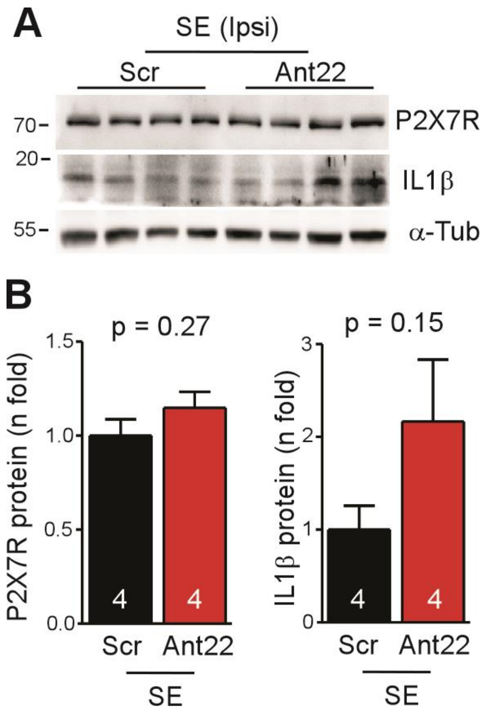

Supplementary data Figure S4. *Acute ipsilateral changes in P2X7R and IL1β levels in antagomir vs scrambled-treated mice.* **(A)** Representative western blots (*n* = 1/lane) show P2X7R protein and IL-1β protein levels in the ipsilateral hippocampus 12 h after SE in mice previously treated with either Ant22 or the scrambled oligo.**(B)** Graphs quantifying protein levels. Note, non-significant trend to increase for both proteins when miR-22 was silenced. Group sizes indicated on individual bars.

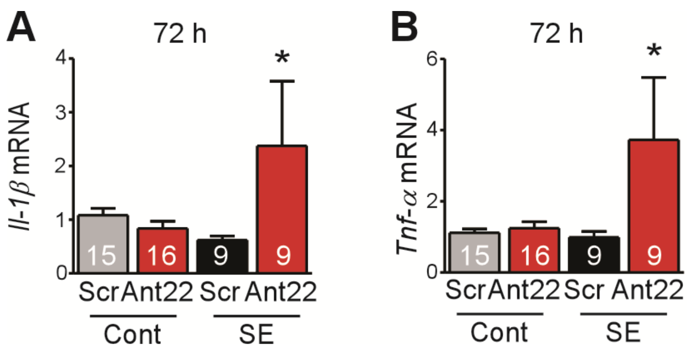

Supplementary data Figure S5. *Increased markers of neuroinflammation in the contralateral hippocampus in Ant22 mice at 72 h.* **(A, B)** Graphs showing increased mRNA levels of the inflammatory genes *Il-1β* and *Tnf-α* in the contralateral hippocampus of Ant22 mice 72 h after SE (latent period). Group sizes indicated on individual bars. * $p < 0.05$ compared to Scr. Cont, non-seizure control.

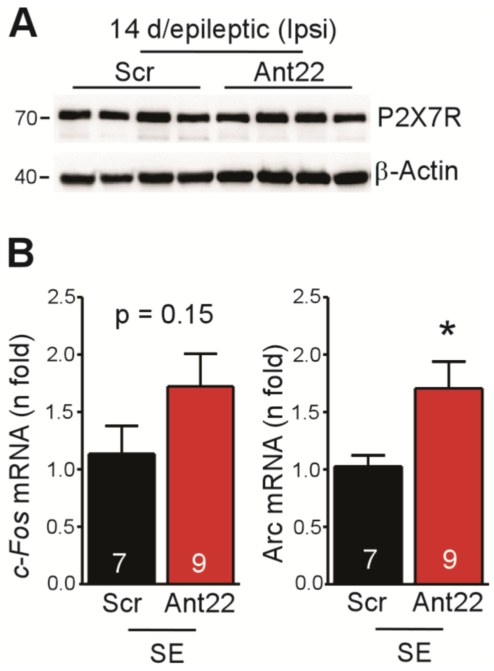

Supplementary data Figure S6. *Chronic ipsilateral changes in P2X7R levels and markers of excitability in antagomir vs scrambled-treated mice.* **(A)** Representative western blots (*n* = 1/lane) showing P2X7R protein levels were similar in the ipsilateral hippocampus at the end of two weeks telemetry recordings in mice previously treated with either Ant22 or the scrambled oligo before SE. **(B)** RT-qPCR data for activity-regulated genes *c-Fos* and *Arc*, surrogate markers of epileptic activity, in the ipsilateral hippocampus at the end of two weeks telemetry recordings in mice previously treated with either Ant22 or the scrambled olio before SE. Group sizes indicated on individual bars.*p < 0.05 compared to Scr.

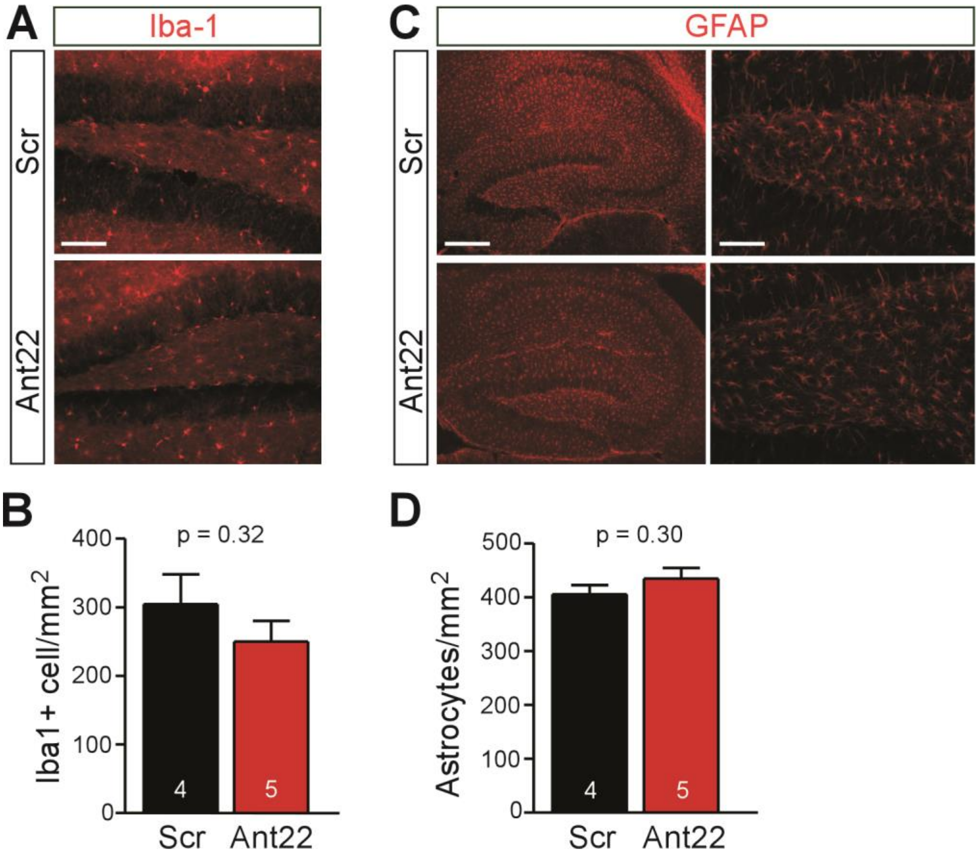

Supplementary data Figure S7. *Chronic ipsilateral changes in Iba1 and GFAP-positive cells in antagomir vs scrambled-treated mice subject to status epilepticus.* **(A)** Representative immunostaining showing Iba1-labeled microglia cells in the ipsilateral hippocampus at the end of two weeks telemetry recordings in mice previously treated with either Ant22 or the scrambled oligo before SE. **(B)** Graph showing similar counts of Iba1-positive cells between the two groups in the ipsilateral hippocampus. **(C)** Representative immunostaining showing GFAP-labeled astrocytes in the ipsilateral hippocampus at the end of two weeks telemetry recordings in mice previously treated with either Ant22 or the scrambled oligo before SE. **(D)** Graph showing similar astrocyte counts between groups on the ipsilateral side. Group sizes indicated on individual bars. Scale bars A, 150 µm; C, left, 1mm; right, 200 µm.
